# 2-(3-Amino­pyridinium-1-yl)-3-carb­oxy­propano­ate monohydrate

**DOI:** 10.1107/S1600536812006897

**Published:** 2012-02-24

**Authors:** Guadalupe Millán Corrales, David Morales-Morales, Simón Hernández-Ortega, José J. Campos-Gaxiola, Adriana Cruz Enríquez

**Affiliations:** a Facultad de Ingeniería Mochis, Universidad Autónoma de Sinaloa, Fuente de Poseidón y Prol. Angel Flores, CP 81223, Los Mochis, Sinaloa, Mexico; b Instituto de Química, Universidad Nacional Autónoma de México, Circuito Exterior, Ciudad Universitaria, Coyoacan, CP 04510, México, DF, Mexico

## Abstract

The title compound, C_9_H_10_N_2_O_4_·H_2_O, was obtained as a zwitterion derived from the nucleophilic attack of 3-amino­pyridine on the fumaric α,β-system. Within the molecule, the amino­pyridine moiety and the carboxyl­ate and carb­oxy­lic acid fragments form dihedral angles of 68.6 (2) and 62.8 (2)°, respectively. The geometry adopted by the mol­ecule does not allow the formation of centrosymmetric dimeric hydrogen-bonded units; instead chains along the *a* axis are linked by COO—H⋯OOC motifs. These chains are inter­connected by N—H⋯O and O—H⋯O hydrogen bonds involving the carb­oxy­lic acid and carboxyl­ate units and the solvent water mol­ecules.

## Related literature

For background to the synthesis, see: Kavuru *et al.* (2010 ▶). For structures and applications of zwitterion derivatives, see: Bis & Zaworotko (2005 ▶); Hill *et al.* (2001 ▶); Sarma *et al.* (2009 ▶). For fundamental hydrogen-bond inter­actions, see: Desiraju (1995 ▶); Etter (1990 ▶, 1991 ▶).
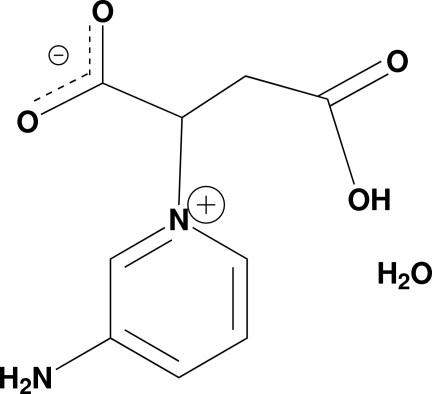


## Experimental

### 

#### Crystal data

C_9_H_10_N_2_O_4_·H_2_O
*M* *_r_* = 228.21
Orthorhombic, 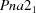

*a* = 7.4939 (8) Å
*b* = 19.446 (2) Å
*c* = 7.0227 (7) Å
*V* = 1023.39 (19) Å^3^
*Z* = 4
Mo *K*α radiation
μ = 0.12 mm^−1^
*T* = 298 K
0.30 × 0.12 × 0.10 mm

#### Data collection

Bruker SMART APEX CCD area-detector diffractometer
10663 measured reflections
1868 independent reflections
1642 reflections with *I* > 2σ(*I*)
*R*_int_ = 0.037

#### Refinement

*R*[*F* ^2^ > 2σ(*F* ^2^)] = 0.028
*wR*(*F* ^2^) = 0.058
*S* = 0.96
1868 reflections
160 parameters
6 restraints
H atoms treated by a mixture of independent and constrained refinement
Δρ_max_ = 0.11 e Å^−3^
Δρ_min_ = −0.12 e Å^−3^

### 

Data collection: *SMART* (Bruker, 2007 ▶); cell refinement: *SAINT* (Bruker, 2007 ▶); data reduction: *SAINT*; program(s) used to solve structure: *SHELXS97* (Sheldrick, 2008 ▶); program(s) used to refine structure: *SHELXL97* (Sheldrick, 2008 ▶); molecular graphics: *ORTEP-3* (Farrugia, 1997 ▶) and *SHELXL97* (Sheldrick, 2008 ▶); software used to prepare material for publication: *SHELXL97*.

## Supplementary Material

Crystal structure: contains datablock(s) I, global. DOI: 10.1107/S1600536812006897/fj2513sup1.cif

Structure factors: contains datablock(s) I. DOI: 10.1107/S1600536812006897/fj2513Isup2.hkl

Supplementary material file. DOI: 10.1107/S1600536812006897/fj2513Isup3.cml

Additional supplementary materials:  crystallographic information; 3D view; checkCIF report

## Figures and Tables

**Table 1:** Hydrogen-bond geometry (Å, °)

*D*—H⋯*A*	*D*—H	H⋯*A*	*D*⋯*A*	*D*—H⋯*A*
O3—H3⋯O2^i^	0.87 (1)	1.60 (1)	2.4681 (15)	177 (2)
O5—H5*A*⋯O4^ii^	0.85 (1)	2.04 (1)	2.8879 (18)	174 (2)
O5—H5*B*⋯O1^iii^	0.86 (1)	1.94 (1)	2.7968 (19)	173 (2)
N2—H2*A*⋯O5^iv^	0.91 (1)	2.02 (1)	2.920 (2)	168 (2)
N2—H2*B*⋯O4^iv^	0.91 (1)	2.08 (1)	2.987 (2)	173 (2)

Symmetry codes: (i) 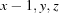; (ii) 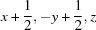; (iii) 
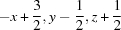
; (iv) 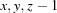.

**Fig. 1.**
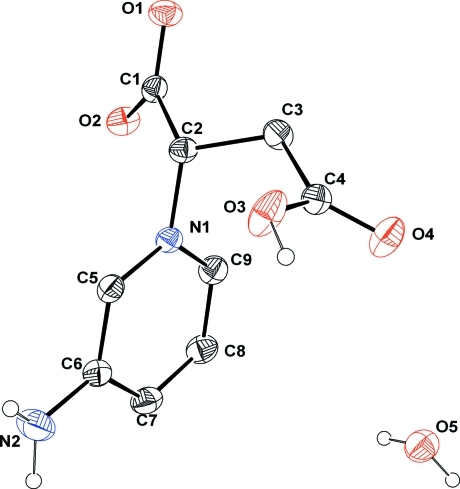
The molecular structure of the title compound (I), with atom labels and displacement ellipsoids drawn at the 40% probability level.

**Fig. 2.**
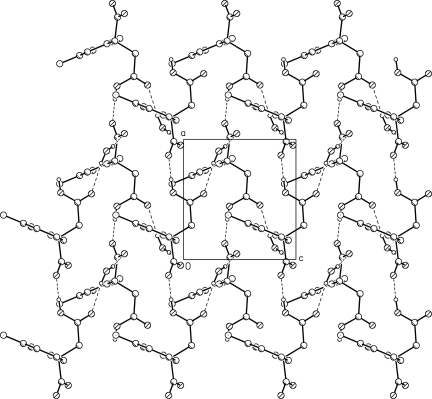
Crystal packing of the title compound (I). Only H atoms involved in interactions were drawn.

## supplementary crystallographic information

### Comment

Crystal engineering is defined in terms of structural geometry and topology (Desiraju, 1995) and hydrogen's rules (Etter, 1990, 1991) are described for predicting hydrogen-bond patterns. Recently, zwitterion derivatives have been of particular interest as building blocks for the synthesis of salts, co-crystals (Kavuru *et al.* 2010) and polymeric compounds (Hill *et al.* 2001). For this purpose, hydrogen bonded supramolecular synthons are commonly used to build co-crystals or organic-based self-assembled structures because of their strength and directionality. In this context, aminopyridines and carboxylic acids have been employed for the generation of multicomponent crystals (Bis & Zaworotko, 2005; Sarma *et al.*, 2009). Thus, in this opportunity we would like to describe the molecular and crystal structure of 2-(3-aminopyridinium) succinate acid monohydrate (I).

The asymmetric unit of I contains one 2-(3-aminopyridinium) succinic acid and one water molecule (Figure 1). The 3-aminopyridinium and the di-acid fragment are not coplanar, and are forming dihedral angles of 68.6 (2)° between the aminopyridinium and the carboxylate anion and of 62.8 (2)° between the 3-aminopyridinium fragment and the carboxylic acid group. The carboxylate anion and carboxylic acid fragments are rotated around C2 and C3 respectively, forming an almost perpendicular dihedral angle of 80.6 (1)°. This adopted geometry does not allow the classical dimers as patterns, and form chains along the *a* axis, *via* O3—H3—O2. These chains are linked by intermolecular interactions of N2—H2B—O4 thus generating a two-dimensional network along the *c* axis (Figure 2). The two-dimensional network is interconnected through O5—H5A—O4, O5—H5B—O1 and N2—HA—O5 hydrogen bonds to give an overall three-dimensional hydrogen bonded network (Table 1 and Fig. 2).

### Experimental

A solution of fumaric acid (0.05 g, 430 mmol) in MeOH (5 ml) was mixed at room temperature for 10 min. After this time 3-aminopyridine (0.04 g, 430 mmol) was added and mixed for further 30 minutes. Crystals suitable for single-crystal *X* ray diffraction studies were grown by slow evaporation at room temperature from a saturated solution of compound (I) in methanol (yield: 60%) m.p. 417 K. IR (KBr): 3414, 3347, 3235, 2581, 2147, 1722, 1652, 1173 *y* 1090 cm^-1^.

### Refinement

H atoms on O and N atoms,were located in the Fourier map and refined isotropically (O—H 0.85 Å, N—H, 0.90 Å). All H atoms were included in calculated positions (C—H = 0.93Å for arom, 0.98Å for methine, 0.97Å for methylene), and refined using a riding model,with *U*_iso_(H) = 1.2*U*_eq_ of the carrier atom. The absolute configuration was not determined by difraction experiment and the enantiomer refined was fixed arbitrary. 852 Friedel pairs were merged.

### Crystal data

**Table:**

C_9_H_10_N_2_O_4_·H_2_O	*F*(000) = 480
*M**_r_* = 228.21	*D*_x_ = 1.481 Mg m^−^^3^
Orthorhombic, *P**n**a*2_1_	Mo *K*α radiation, λ = 0.71073 Å
Hall symbol: P 2c -2n	Cell parameters from 5172 reflections
*a* = 7.4939 (8) Å	θ = 2.9–25.3°
*b* = 19.446 (2) Å	µ = 0.12 mm^−^^1^
*c* = 7.0227 (7) Å	*T* = 298 K
*V* = 1023.39 (19) Å^3^	Prism, orange
*Z* = 4	0.30 × 0.12 × 0.10 mm

### Data collection

**Table:**

Bruker SMART APEX CCD area-detector diffractometer	1642 reflections with *I* > 2σ(*I*)
Radiation source: fine-focus sealed tube	*R*_int_ = 0.037
Graphite monochromator	θ_max_ = 25.3°, θ_min_ = 2.1°
Detector resolution: 0.83 pixels mm^-1^	*h* = −9→9
ω scans	*k* = −23→23
10663 measured reflections	*l* = −8→8
1868 independent reflections	

### Refinement

**Table:**

Refinement on *F*^2^	Primary atom site location: structure-invariant direct methods
Least-squares matrix: full	Secondary atom site location: difference Fourier map
*R*[*F*^2^ > 2σ(*F*^2^)] = 0.028	Hydrogen site location: inferred from neighbouring sites
*wR*(*F*^2^) = 0.058	H atoms treated by a mixture of independent and constrained refinement
*S* = 0.96	*w* = 1/[σ^2^(*F*_o_^2^) + (0.028*P*)^2^] where *P* = (*F*_o_^2^ + 2*F*_c_^2^)/3
1868 reflections	(Δ/σ)_max_ < 0.001
160 parameters	Δρ_max_ = 0.11 e Å^−^^3^
6 restraints	Δρ_min_ = −0.12 e Å^−^^3^

### Special details

**Table:**

Geometry. All e.s.d.'s (except the e.s.d. in the dihedral angle between two l.s. planes) are estimated using the full covariance matrix. The cell e.s.d.'s are taken into account individually in the estimation of e.s.d.'s in distances, angles and torsion angles; correlations between e.s.d.'s in cell parameters are only used when they are defined by crystal symmetry. An approximate (isotropic) treatment of cell e.s.d.'s is used for estimating e.s.d.'s involving l.s. planes.
Refinement. Refinement of *F*^2^ against ALL reflections. The weighted *R*-factor *wR* and goodness of fit *S* are based on *F*^2^, conventional *R*-factors *R* are based on *F*, with *F* set to zero for negative *F*^2^. The threshold expression of *F*^2^ > σ(*F*^2^) is used only for calculating *R*-factors(gt) *etc*. and is not relevant to the choice of reflections for refinement. *R*-factors based on *F*^2^ are statistically about twice as large as those based on *F*, and *R*- factors based on ALL data will be even larger.

### Fractional atomic coordinates and isotropic or equivalent isotropic displacement parameters (Å^2^)

**Table:**

	*x*	*y*	*z*	*U*_iso_*/*U*_eq_	
O1	1.04824 (15)	0.51597 (6)	0.4732 (2)	0.0417 (3)	
O2	1.13193 (13)	0.41287 (5)	0.36700 (19)	0.0395 (3)	
O3	0.44407 (15)	0.45071 (7)	0.4070 (2)	0.0565 (4)	
H3	0.3347 (15)	0.4376 (9)	0.389 (3)	0.068*	
O4	0.45279 (16)	0.39455 (7)	0.67937 (19)	0.0528 (4)	
O5	0.59669 (18)	0.13525 (7)	0.8132 (2)	0.0557 (4)	
H5A	0.6976 (17)	0.1248 (11)	0.767 (3)	0.067*	
H5B	0.559 (3)	0.0989 (7)	0.870 (3)	0.067*	
N1	0.79643 (15)	0.36728 (7)	0.3184 (2)	0.0291 (3)	
N2	0.6301 (2)	0.28176 (8)	−0.1026 (2)	0.0554 (4)	
H2A	0.605 (2)	0.2377 (6)	−0.137 (3)	0.066*	
H2B	0.581 (2)	0.3184 (8)	−0.163 (3)	0.066*	
C1	1.0183 (2)	0.45789 (8)	0.4126 (2)	0.0307 (4)	
C2	0.81973 (19)	0.43883 (7)	0.3871 (2)	0.0299 (4)	
H2	0.7699	0.4696	0.2902	0.036*	
C3	0.7156 (2)	0.45043 (9)	0.5703 (3)	0.0363 (4)	
H3A	0.7718	0.4246	0.6721	0.044*	
H3B	0.7217	0.4988	0.6036	0.044*	
C4	0.5235 (2)	0.42955 (8)	0.5568 (3)	0.0341 (4)	
C5	0.7281 (2)	0.35719 (8)	0.1437 (2)	0.0325 (4)	
H5	0.6999	0.3950	0.0682	0.039*	
C6	0.6988 (2)	0.29126 (9)	0.0739 (3)	0.0359 (4)	
C7	0.7427 (2)	0.23635 (9)	0.1932 (3)	0.0411 (5)	
H7	0.7242	0.1914	0.1522	0.049*	
C8	0.8125 (2)	0.24828 (8)	0.3696 (3)	0.0412 (4)	
H8	0.8415	0.2114	0.4481	0.049*	
C9	0.8403 (2)	0.31445 (8)	0.4320 (3)	0.0376 (4)	
H9	0.8891	0.3225	0.5517	0.045*	

### Atomic displacement parameters (Å^2^)

**Table:**

	*U*^11^	*U*^22^	*U*^33^	*U*^12^	*U*^13^	*U*^23^
O1	0.0332 (7)	0.0403 (7)	0.0515 (8)	−0.0082 (5)	−0.0007 (6)	−0.0106 (6)
O2	0.0239 (6)	0.0369 (6)	0.0577 (8)	0.0034 (5)	0.0002 (6)	−0.0013 (6)
O3	0.0244 (7)	0.0849 (10)	0.0600 (9)	−0.0104 (6)	−0.0076 (8)	0.0293 (8)
O4	0.0381 (8)	0.0673 (9)	0.0530 (9)	−0.0094 (6)	−0.0001 (7)	0.0195 (8)
O5	0.0528 (8)	0.0420 (7)	0.0724 (11)	0.0014 (7)	0.0116 (8)	−0.0036 (8)
N1	0.0218 (6)	0.0325 (7)	0.0330 (8)	−0.0018 (6)	0.0001 (6)	−0.0012 (6)
N2	0.0796 (12)	0.0400 (9)	0.0466 (11)	−0.0056 (9)	−0.0157 (10)	−0.0030 (9)
C1	0.0278 (9)	0.0351 (9)	0.0293 (9)	−0.0034 (7)	−0.0018 (8)	0.0034 (8)
C2	0.0243 (8)	0.0306 (8)	0.0349 (10)	−0.0011 (6)	−0.0011 (8)	−0.0015 (8)
C3	0.0266 (9)	0.0443 (10)	0.0380 (10)	−0.0004 (8)	−0.0001 (8)	−0.0074 (8)
C4	0.0261 (9)	0.0367 (9)	0.0394 (11)	0.0021 (8)	0.0020 (9)	−0.0027 (9)
C5	0.0304 (9)	0.0339 (9)	0.0330 (11)	−0.0031 (7)	0.0021 (8)	0.0031 (8)
C6	0.0346 (9)	0.0382 (10)	0.0351 (11)	−0.0071 (8)	−0.0010 (9)	−0.0009 (9)
C7	0.0414 (11)	0.0306 (9)	0.0512 (13)	−0.0046 (8)	0.0031 (10)	−0.0005 (9)
C8	0.0428 (10)	0.0336 (9)	0.0472 (12)	−0.0006 (8)	−0.0036 (10)	0.0065 (9)
C9	0.0327 (9)	0.0421 (10)	0.0380 (11)	−0.0026 (8)	−0.0042 (9)	0.0030 (8)

### Geometric parameters (Å, º)

**Table:**

O1—C1	1.2276 (18)	C2—C3	1.522 (2)
O2—C1	1.2623 (19)	C2—H2	0.9800
O3—C4	1.277 (2)	C3—C4	1.499 (2)
O3—H3	0.868 (9)	C3—H3A	0.9700
O4—C4	1.2183 (19)	C3—H3B	0.9700
O5—H5A	0.848 (9)	C5—C6	1.390 (2)
O5—H5B	0.861 (9)	C5—H5	0.9300
N1—C9	1.341 (2)	C6—C7	1.396 (2)
N1—C5	1.344 (2)	C7—C8	1.365 (3)
N1—C2	1.4830 (18)	C7—H7	0.9300
N2—C6	1.355 (2)	C8—C9	1.375 (2)
N2—H2A	0.909 (9)	C8—H8	0.9300
N2—H2B	0.907 (9)	C9—H9	0.9300
C1—C2	1.544 (2)		
			
C4—O3—H3	117.7 (16)	C2—C3—H3B	108.9
H5A—O5—H5B	106 (2)	H3A—C3—H3B	107.7
C9—N1—C5	121.62 (14)	O4—C4—O3	124.03 (15)
C9—N1—C2	119.75 (14)	O4—C4—C3	121.62 (16)
C5—N1—C2	118.62 (13)	O3—C4—C3	114.34 (16)
C6—N2—H2A	116.6 (14)	N1—C5—C6	121.11 (15)
C6—N2—H2B	118.2 (14)	N1—C5—H5	119.4
H2A—N2—H2B	122.2 (18)	C6—C5—H5	119.4
O1—C1—O2	127.07 (14)	N2—C6—C5	120.55 (16)
O1—C1—C2	115.91 (14)	N2—C6—C7	122.28 (17)
O2—C1—C2	117.02 (14)	C5—C6—C7	117.17 (17)
N1—C2—C3	110.72 (13)	C8—C7—C6	120.32 (16)
N1—C2—C1	112.11 (12)	C8—C7—H7	119.8
C3—C2—C1	111.15 (13)	C6—C7—H7	119.8
N1—C2—H2	107.5	C7—C8—C9	120.42 (17)
C3—C2—H2	107.5	C7—C8—H8	119.8
C1—C2—H2	107.5	C9—C8—H8	119.8
C4—C3—C2	113.52 (14)	N1—C9—C8	119.35 (16)
C4—C3—H3A	108.9	N1—C9—H9	120.3
C2—C3—H3A	108.9	C8—C9—H9	120.3
C4—C3—H3B	108.9		
			
C9—N1—C2—C3	−57.28 (17)	C2—C3—C4—O3	−46.5 (2)
C5—N1—C2—C3	121.47 (14)	C9—N1—C5—C6	0.5 (2)
C9—N1—C2—C1	67.48 (17)	C2—N1—C5—C6	−178.25 (13)
C5—N1—C2—C1	−113.77 (15)	N1—C5—C6—N2	−179.88 (16)
O1—C1—C2—N1	−178.48 (14)	N1—C5—C6—C7	0.3 (2)
O2—C1—C2—N1	2.4 (2)	N2—C6—C7—C8	179.63 (17)
O1—C1—C2—C3	−53.95 (19)	C5—C6—C7—C8	−0.5 (3)
O2—C1—C2—C3	126.92 (16)	C6—C7—C8—C9	0.1 (3)
N1—C2—C3—C4	−51.92 (18)	C5—N1—C9—C8	−0.9 (2)
C1—C2—C3—C4	−177.22 (13)	C2—N1—C9—C8	177.77 (14)
C2—C3—C4—O4	132.57 (18)	C7—C8—C9—N1	0.7 (3)

### Hydrogen-bond geometry (Å, º)

**Table:**

*D*—H···*A*	*D*—H	H···*A*	*D*···*A*	*D*—H···*A*
O3—H3···O2^i^	0.87 (1)	1.60 (1)	2.4681 (15)	177 (2)
O5—H5*A*···O4^ii^	0.85 (1)	2.04 (1)	2.8879 (18)	174 (2)
O5—H5*B*···O1^iii^	0.86 (1)	1.94 (1)	2.7968 (19)	173 (2)
N2—H2*A*···O5^iv^	0.91 (1)	2.02 (1)	2.920 (2)	168 (2)
N2—H2*B*···O4^iv^	0.91 (1)	2.08 (1)	2.987 (2)	173 (2)

Symmetry codes: (i) *x*−1, *y*, *z*; (ii) *x*+1/2, −*y*+1/2, *z*; (iii) −*x*+3/2, *y*−1/2, *z*+1/2; (iv) *x*, *y*, *z*−1.
